# Relationship between Biofilm Production and High Somatic Cell Count in *Streptococcus agalactiae* Isolated from Milk of Cows with Subclinical Mastitis

**DOI:** 10.3390/pathogens12020311

**Published:** 2023-02-14

**Authors:** Erika Carolina Romão Bonsaglia, Rodolfo S. Rossi, Giulia Latosinski, Bruna Fernanda Rossi, Fernanda Cristina Campos, Ary Fernandes Junior, José Carlos F. Pantoja, Vera Lucia Mores Rall

**Affiliations:** 1 Department of Chemical and Biological Sciences, Institute of Biosciences, Sao Paulo State University (UNESP), Botucatu 18618-970, SP, Brazil; 2 Department of Veterinary Hygiene and Public Health, Sao Paulo State University (UNESP), Botucatu 18618-970, SP, Brazil

**Keywords:** *Streptococcus agalactiae*, mastitis, capsular type, biofilm, SCC

## Abstract

*Streptococcus agalactiae* (*S. agalactiae*) is one of the main agents that causes mastitis in dairy cows, mainly inducing the subclinical form, which is characterized by a high somatic cell count (SCC). The aim of this study was to correlate the increase in SCC caused by *S. agalactiae* in cows with subclinical mastitis to the presence of genes related to adhesion and invasion in bovine mammary epithelial cells (BMEC) and biofilm formation. Considering the 145 isolates tested, 57.2% presented the capsular type Ia and 42.8% presented type III. We identified the virulence genes among the isolates and determined nine genetic profiles. The most common profile was identified in 69 isolates (47.5%): *Ia*, *fbsA*^+^, *fbsB*^-^, *pI1*^-^, *pI2a*^-^, *pI2b*^+^, and *hylb*^+^. All isolates produced biofilm, with 58.6% classified as strong producers, 29% as moderate producers and 12.4% as weak producers. No statistical correlation was found between the presence of virulence genes and increased SCC or biofilm production. However, biological evidence was observed between increased SCC and biofilm production. One isolate from each profile was randomly subjected to adhesion and invasion assays, and all of them adhered to BEMC, but none were able to invade. Our results showed that different genetic profiles do not provide advantages for bacteria to invade BMEC in vitro. In addition, biofilm production appears to be related to high SCC.

## 1. Introduction

*Streptococcus agalactiae* has been considered one of the most common etiological agents of intramammary infection in dairy cows, causing both clinical and subclinical mastitis [1]. In the last decade, *S. agalactiae* has been isolated from herds in several countries around the world, including Italy, Colombia, China, Poland, and Denmark [2,3,4,5,6], and has been detected in 60% of herds in Brazil [7]. Over the years, *S. agalactiae* has been recognized as an obligate parasite of the mammary gland [8], but recent studies have demonstrated the ability of this pathogen to survive in extramammary environments, such as the bovine gastrointestinal tract and the milking environment (cow beds, floors, and water channels), suggesting a transmission cycle that includes infected mammary glands and contaminated environmental sources, such as the milking machine, bedding, and water [3,9]. This micro-organism primarily infects the cistern and lactiferous ducts of the mammary gland, where its associated metabolic products elicit an intense inflammatory response; this response results in tissue damage, reduced milk yield, and a geometric mean in the somatic cell count (SCC) of 857,000 cells/ml in infected cow’s milk [10]. It is the costliest disease of dairy cows, and the most significant economic losses occur from reduced milk production and costs associated with therapy [7]. Furthermore, *S. agalactiae* is a public health concern; studies have shown an association between raw milk consumption and an increased risk of *S. agalactiae* infection in humans. The authors demonstrated, by means of molecular characterization, that human samples associated with neonatal disease were of bovine origin [11,12]. The study of Carra et al. [2] also supported the hypothesis of *S. agalactiae* transmission between cattle and humans, and Botelho et al. [13] suggested that it can occur during milking, consumption of raw, contaminated milk, or by means of environmental contamination.

Capsular polysaccharide (CPS) search is the main method used to characterize the species and is an essential virulence factor of *S. agalactiae* that impairs the host’s recognition of the pathogen and is composed of sialic acid (commonly found in vertebrate glycans) [14]. CPSs are classified into 10 serotypes (Ia, Ib, and II-IX), with the Ia, Ib, III, IV, and V types being the most common in mastitis isolates. The pathogenicity of *S. agalactiae* varies with its serotypes, although its distribution seems to be related to the geographical region [7,14,15]. Even in the same country, a diverse prevalence rate can be found among regions, as shown by Carvalho-Castro et al. [7], who compared strains from the North, South, and Southeast regions of Brazil, demonstrating a difference between the capsular types of bovine isolates. This observation may indicate the occurrence of capsular switching in *S. agalactiae*, driven by the host immune response, an event that has been well documented in other streptococcal species [16]. Furthermore, studies have shown that CPS plays an important role in Group B streptococcus (GBS) adherence and invasion of cells, as well as contributing to biofilm formation [17]. 

Biofilm represents a bacterial mode of protection, and its presence has effects on many branches of the dairy environment. Damage is related to the contamination of raw milk pipelines, cooling milk tanks, and milking machines that affect udder health, and therefore, reduce milk quality [18]. Recent advances investigating the potential of *Staphylococcus aureus* to form biofilms in the mammary gland and induce distinct immune responses in mouse mastitis models may help to elucidate the complex relationship between biofilm and the host immune system [19,20]. These studies encourage further research on other pathogens that cause mastitis in cattle because it is already known that biofilms can activate the immune system in a unique way depending on the bacteria involved and the immune status of the host [21]. 

Biofilm formation by *S. agalactiae* has been studied in vitro and increasingly appears to be controlled by environmental conditions [22]. Studies have shown that host mammary cells´ viability is suddenly reduced after 2 h of infection with this pathogen and that bacterial adhesion is achieved after 6 h of infection [14]. Adhesion to host tissues is the initial event in bacterial pathogenesis, and *S. agalactiae* uses multiple adhesins to attach to the epithelium. The recognition of cell surface molecules and extracellular matrix components through specific domains and structures, such as pili, are considered precursors in the infection process [23]. In addition, the pili of *S. agalactiae* have been shown to promote the invasion of human endothelial cells and influence biofilm formation [22]. These structures are cell-wall appendages extending from the bacterial surface that were discovered during multigenome screening for surface proteins exposed to antigens as possible vaccine targets and are encoded by two loci designated as pilus islands 1 and 2 (PI-1, PI-2), the latter presenting two variants, PI-2a and PI-2b [24,25]. According to previous reports, the pilus may play a role in the host specificity of *S. agalactiae*, as PI-2a is highly correlated with human-derived isolates and PI-2b with animal-derived isolates [26,27].

Other virulence factors, such as the capacity to interact with extracellular matrix fibrinogen, are mediated by two proteins known as FbsA and FbsB, which are responsible for adhesion and invasion of host cells, and the hylB protein (hyaluronate lyase). This protein is responsible for cleaving hyaluronan, which facilitates the propagation of *S. agalactiae* during infection [28]. Based on current knowledge, the role of *S. agalactiae* biofilm in bovine mastitis is still unclear, and more in vitro and in vivo investigations are needed to uncover the relationship between *S. agalactiae biofilm* and the host immune system. 

We, therefore, hypothesized that there is a relationship between the high SCC caused by *S. agalactiae* in cows with subclinical mastitis and biofilm formation and the presence of virulence genes related to adherence and invasion of bovine mammary epithelial cells (BMEC). Thus, the aim of this study was to correlate the increase in SCC caused by *S. agalactiae* in cows with subclinical mastitis to the presence of genes related to adhesion and invasion in bovine mammary epithelial cells (BMEC) and biofilm formation.

## 2. Material and Methods

### 2.1. Bacterial Isolates and Molecular Typing

This study was approved by the Sao Paulo State University (UNESP) Ethics Committee for Animal Use (protocol 07/2015). The one hundred and forty-five isolates used in this study were isolated from milk samples of cows diagnosed with subclinical *S. agalactiae* mastitis between February and October 2016; the cows were from nine farms located in the states of Sao Paulo and Minas Gerais, Brazil [29]. Milk samples were collected from all mammary quarters of all lactating cows as part of a herd screening plan. After the milking technicians performed the pre-milking hygiene procedures (visual examination of the first milk streams to detect clinical mastitis, pre-dipping with iodine or chlorine solutions, and drying of teats with a paper towel), the test end was disinfected with a cotton ball soaked in alcohol 70%. Subsequently, three milk streams were discarded, and 15 mL of milk were collected by manual milking into a sterile plastic vial. Samples were frozen after collection and transported to the laboratory. 

Isolates were identified as *S. agalactiae* if they were gram-positive cocci, esculin- and bile-esculin negative, catalase-negative, and positive for the Christie, Atkins, Munch-Petersen (CAMP) test. All the isolates were further submitted to PCR to confirm the identification.

The DNA from bacterial cells was extracted using the MiniSpin Kit (GE Healthcare, Little Chafont, Buckinghamshire), according to the manufacturer’s instructions. GoTaq 2× Green Master Mix (Promega, Madison, WI, USA) was also used in the polymerase chain reaction (PCR) as recommended, and the assays were performed in a Gene Amp PCR System9700 (Applied Biosystems, Carlsbad, CA, USA). Molecular confirmation was performed using species-specific primers for *S. agalactiae* (*A2* gene), according to Almeida et al. [30]. Following identification, PCR was performed to determine the capsular types for the *Ia, Ib,* and *II-IX* genes, as described by Poyart et al. [15]. The presence of *pI1, pI2a, and pI2b* (involved in host cell invasion and biofilm formation) [16,31], and *fbsA, fbsB, and hylB* (involved in host cell adhesion) was also investigated [32]. Sanger sequencing was performed on one strain that was positive for each gene and used as a positive control.

Somaticell (Idexx Laboratories Inc., Westbrook, ME, USA) was used to estimate SCC, and was performed using refrigerated milk upon arrival at the laboratory, following the manufacturer’s instructions. Two milliliters of reagent and milk were mixed in a plastic tube and homogenized for 30 s. The tube was then held upside down for 30 s to drain the solution, and the final reading (cells/mL) was performed using the tube scale. The range of SCC for *S. agalactiae* used in this study was previously described by Rossi et al. [29]. 

### 2.2. Biofilm Production 

Biofilm production was performed according to Bonsaglia et al. [33]. An overnight growth of *S. agalactiae*, in tryptone soy broth (TSB, Oxoid, Basingstoke, United Kingdom) was adjusted to the 0.5 McFarland standard, and aliquots of 200 µL were distributed, in quadruplicate, into 96-well polystyrene microplates (TPP, Trasadingen, Switzerland) and incubated at 37 °C for 18 h in a 5% CO_2_ atmosphere. 

After washing three times in sterile phosphate buffered saline (PBS, pH 7.2), the wells were stained with 0.1% crystal violet for 30 minutes. Subsequently, they were rinsed with PBS and left to dry at room temperature. Following, 200 μL of 33% acetic acid was added to each well for biofilm detachment and homogenization. An optical density (OD) of 595 nm was used to measure the wells using a microplate reader (Epoch 2 Microplate Reader, Biotek, Winooski, VT, USA). Uninoculated wells containing TSB were used as blanks to correct absorbance values. Biofilm quantification was performed according to Stépanovic et al. [34], classifying the isolates as: nonbiofilm producers (NP), strong biofilm producers (SBP), moderate biofilm producers (MBP), and weak biofilm producers (WBP). This was based upon the previously calculated OD values: (for this type of calculation, the average OD value of the strain should not be reduced by the optical density control value, ODc) OD ≤ ODc = no biofilm producer; ODc < OD ≤ 2× ODc = weak biofilm producer; 2× ODc < OD ≤ 4× ODc = moderate biofilm producer; 4× ODc < OD = strong biofilm producer.

### 2.3. Adhesion and Invasion Assays for BMEC Cells

Assays were performed according to Sharma et al. [35], with modifications. BMEC cells were cultured in 24-well microplates, and BMEC monolayers were infected, in duplicate, with *S. agalactiae* cultures diluted in Dulbecco modified eagle’s medium (DMEM) (1.5 × 10^6^ CFU/mL); and 1 ml of these suspensions were inoculated into each well and incubated at 37 °C in a 5% CO_2_ atmosphere. Following 3 h of incubation, infected monolayers were washed three times with PBS to remove non-adherent bacterial cells, and 500 μL of trypsin-EDTA (0.1%/0.04%) was added to detach the cells; this was followed by incubation for 15 minutes in 5% CO_2_. After that, a 500-μL aliquot of DMEM was mixed, and serial dilutions in saline solutions were created. Then, 10 mL were seeded onto tryptone soya agar (TSA, Oxoid) plates and incubated overnight at 37 °C; *S. agalactiae* CFU were then counted. 

For the invasion test, every condition was kept the same, as mentioned above, until after the first three hours of incubation. At that time, the wells were washed again and incubated for 2 hours in DMEM with 100 μg/mL gentamicin + 5 μg/mL penicillin, to remove the extracellular bacteria. Next, 0.1% Triton X-100 was added to lyse the cells, and 10 μL of a serial dilution were plated onto TSA. A positive result was determined by recovering and counting the CFU in relation to the values obtained in the adhesion test (adhered bacteria/internalized bacteria × 100).

## 3. Results

### 3.1. Genetic Profile

*S. agalactiae* species were confirmed in all isolates carrying the *A2* gene. The *hylB* gene was the most prevalent virulence gene in 97.2% (N = 141/145) of the isolates. Regarding capsular types, Ia was found in 57.2% (N = 83/145) of isolates, followed by type III in 42.7% (N = 62/145) of isolates. Nonetheless, the presence of *fbsA* and *fbsB* among the isolates was 59.3% (N = 86) and 40.6% (N = 59), respectively. PI2b was observed in 95.8% (N = 139) of isolates and pI2a (N = 29) in 20%. PI1 was not found.

According to molecular analysis, all isolates were classified into nine different genetic profiles (Table 1). Out of all isolates, 69 (47.5%) were found to share the same profile, making it the most common. This genetic profile consisted of: capsular type Ia, *fbsA^+^, fbsB^™^, pI1^™^, pI2a^™^, pI2b^+^, and hylb^+^.*

### 3.2. Biofilm Production and SCC the Relationship

We observed biofilm production in all isolates on polystyrene microplates. Eighty-five isolates (58.6%) were classified as SBP (51/85 serotype Ia and 34/85 serotype III), followed by 42 isolates (29%) as MBP (27/42 serotype Ia and 15/42 serotype III), and 18 isolates (12.4%) as WBP (9/18 serotype Ia and 9/18 serotype III).

**Figure 1:**
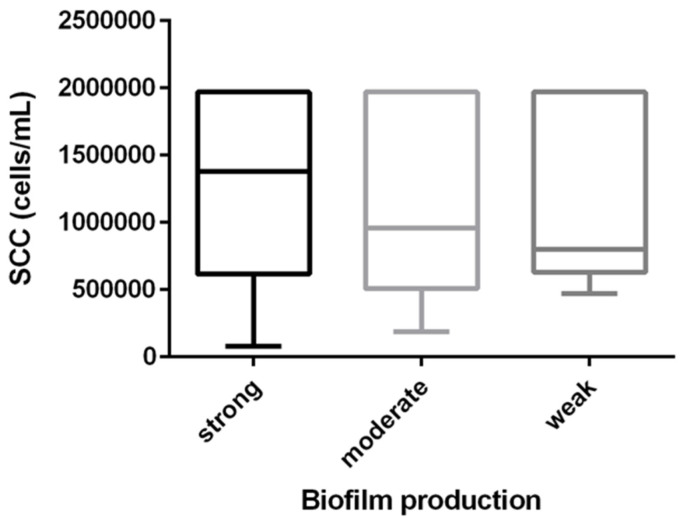
Distribution of SCC in relation to biofilm production by *S. agalactiae* causing bovine mastitis.

Although no statistical difference was observed regarding biofilm production and SCC, the biological trend indicated that SBP triggered a higher SCC response than moderate or weak producers (Figure 1). 

A comparative analysis regarding SCC and biofilm production between virulence genes was also performed, and no correlation was identified in either analysis regarding virulence genes. The data are shown separately in Table 2. 

### 3.3. Cell Adhesion and Invasion 

Considering the nine different profiles observed, only one isolate representing each profile was chosen randomly for the adhesion and invasion assays (Table 1).

A positive result was determined by recovering and counting the colonies in percentages related to the values obtained in the adhesion test. Results showed that all strains adhered to BMEC, but none were able to invade, regardless of the genotypic profile. The values for the adhesion assays are presented in Table 1. The Fisher exact test was used for analysis; however, a valid conclusion was difficult to obtain since some profiles presented too small a sample size. 

### 3.4. Statistical Analysis

Initially, the distribution of the dependent variables, SCC, and biofilm OD was analyzed and transformed to a log10 scale to achieve normality. An analysis of variance (ANOVA, PROC GLM, SAS Institute, Cary, NC, USA) was used to: (1) compare the mean SCC (log10 cells/ml; dependent variable) among isolates that were WBP, MBP, or SBP (independent variable); and (2) compare the mean SCC and OD (dependent variables) among strains that were positive or not for the genes studied (independent variable). The Tukey´s test was used to adjust the P-values resulting from multiple comparisons. The analysis was performed at an alpha level of 0.05. 

## 4. Discussion

Currently, *S. agalactiae* is a common cause of mastitis in dairy herds in many countries [19,20]. The correlation between intramammary infection and a high SCC response (> 600.000 cells/ml) has not yet been fully elucidated; however, our study provides evidence that biofilm production is related to this trait. 

The capsular type is the most studied virulence factor, and the main types we identified were Ia (57%) and III (43%). However, Carvalho-Castro et al. [7] reported five *cps* types isolated from mastitis cases, with type III being the most prevalent (38.9%), followed by type II (20.3%), Ib (15%), Ia (8.4%), and IV (8.4%). In previous studies, the distribution of *S. agalactiae* capsular types depended on geographic location and diagnostic techniques. The fact that there is not a specific *S. agalactiae* strain associated with mastitis may be due to lack of host specificity, as observed by Sorensen et al. [36], who showed that herdspersons and cattle often share the same *S. agalactiae* clone, independent of capsular type. In any case, the capsular type allows bacteria to evade the host immune response [37], and this ability, associated with other virulence factors, contributes to the success of the infection. Another possible reason for the diversity of serotypes observed in bovine mastitis could be due to capsular switching events. Despite the scarce data reported in the literature, some studies suggest that this occurs in *S. agalactiae* [16]. Capsular exchange may confer a survival advantage for bacteria against host-acquired immune responses, although further research is required. According to Paveenkittiporn et al. [38], serotype III is the most associated with a broad spectrum of invasive human infections, as well as this serotype displaying much higher virulence than serotype Ia when infecting fish. In a previous investigation, we showed that some lineages were already adapted to specific host species, but on the other hand, and existed as “core population” of clones, capable of colonizing indistinct hosts. However, this could also be a variation within the same sequence-type (ST), triggering sub-lineages that are still unknown. A limitation of this study was that we were not able to perform sequence typing on our isolates, for further discussion. However, further studies with different serotypes, STs, and hosts may help determine how these host-pathogen interactions act to trigger the emergence of sub-lineages within the clone.

In our study, we also investigated the presence of virulence genes related to adhesion and invasion (*fbsA, fbsB, PI1, PI2a,* and *PI2b*), as well as tissue damage (*hylB*). Fibrinogen binding proteins known as FbsA and FbsB can mediate adherence and promote entry of *S. agalactiae* into host cells, respectively. The results show that 85 isolates carried the *fbsA* gene, followed by 59 isolates that were positive for the *fbsB* gene. Some differences were found by Carvalho-Castro et al. [7], where *fbsA* was present in 42.3% of the isolates and *fbsB* in all of them. Nagao [28] affirmed that even though both were binding proteins, the contributions of their interactions that influence virulence are not well-defined. Regardless of the presence of *hylB,* the prevalence was similar to that observed by Carvalho-Castro et al. [7] in dairy herds in other regions of Brazil. These authors also observed the same prevalence in their isolates as our results, in regards to *pI2b*, with *pI2a* being less prevalent in both studies.

Studies have shown the relationship between pilus loci and biofilm formation in *S. agalactiae* [26]. Here, all isolates were biofilm producers, but the distribution of the presence of pilus island genes (*PI1* and *PI2a* - *PI2b*) was varied, and *pI1* was not observed. Our results agree with those of Alvim et al. [39], who reported that *PI2b* represented 74% of the animal isolates. This may have occurred because each *S. agalactiae* genome encodes one or two distinct pilus islands, and *pI1* has been observed among human isolates [27]. In our study, no statistical correlation was found between virulence genes and biofilm production, nor between virulence genes and SCC.

Pang et al. [27] related that *S. agalactiae* isolates from bovines grew much faster than strains isolated from other sources. This feature supports the fact that infection caused by this pathogen presents high SCC values, since the relationship between SCC and infection occurs because bacteria invade the udder and multiply. Consequently, high bacterial density promotes biofilm formation, causing a persistent and destructive inflammatory process [40]. In general, most knowledge of host immune defense comes from interactions with planktonic cell pathogens, and the response regarding biofilms has not been elucidated [41].

We observed a biological trend suggesting that SBP isolates were associated with higher SCC (Figure 1). The lack of statistical significance between biofilm production and increased SCC may be explained by the extreme SCC variation found among isolates (Figure 1) and the sample size used in the study. Larger studies are necessary to further investigate the SCC response among *S. agalactiae* strains with different levels of biofilm production. Gogoi-Tiwari et al. [19] noted an increase in TNF-α levels in mice infected with a strong biofilm-producing *S. aureus*, compared to a weak biofilm-producing *S. aureus*. In addition, in another study [20], these authors demonstrate that *S. aureus* in a biofilm induces stronger and more differentiated immune responses than its planktonic cells. Despite the lack of studies on *S. agalactiae* in biofilm and immune responses, we can suggest that the presence of *S. agalactiae* biofilm producers promotes the colonization of mammary tissue by the pathogen. This might result in higher bacterial loads and dispersion into the mammary glands, increasing the interaction between pathogens and the immune response. Since SCC is an indicator of the immunological condition of the mammary gland and TNF-α is capable of recruiting macrophages and monocytes [42], we can consider that higher immune system activation may depend on the pathogen’s ability to form a biofilm.

Bacterial pathogenicity engages many mechanisms to cause disease, and the first of them is related to host tissue adhesion. In this study, we also hypothesized that the different genetic profiles of the isolates could show distinct abilities in the establishment of infection. However, we observed that all tested isolates had the same behavior in in vitro adhesion and invasion assays. Curiously, neither of them could invade BMEC, even though the adhesion rate had been similar in all isolates, independent of their genetic profiles. Reduced invasion in *S. agalactiae* can occur due to the production of CPS [43]. In addition, according to Shabayek and Spellerberg [44], invasive *S. agalactiae* strains are more likely to carry a combination of *pI* and one of the *pI*-2 variants, and bovine isolates mostly lack PI1; corroborating our results, where *pI1* was not observed in all isolates. Moreover, it should be noted that the ability to invade BMEC in vitro may not always reflect the in vivo situation. 

## 5. Conclusions

In summary, our results showed that the different genetic profiles presented here do not provide advantages for bacteria to invade BMEC in vitro, and the capsular type showed no correlation with SCC. On the other hand, we demonstrated for the first time that biofilm production is closely related to SCC, considering that activation of the immune response may occur, according to the pathogen’s ability to form biofilm. Thus, the data obtained in our study constitutes a preliminary step for further research to better understand the host-pathogen interaction.

## Figures and Tables

**Table 1:** Distribution of *S. agalactiae* isolated from milk of cows with subclinical mastitis according to genotypic profile, and in adhesion assays with bovine mammary epithelial cells.

Profile	Isolates (n = 145)	serotype	*fbsA*	*fbsB*	*pI1*	*pI2a*	*pI2b*	*hylb*	Adhesion BMEC *
1	69	Ia	+	-	-	-	+	+	1.5
2	11	III	+	-	-	-	+	+	0.3
3	8	Ia	-	+	-	+	-	+	0.4
4	20	III	-	+	-	+	-	+	1.2
5	3	III	-	+	-	-	+	-	1.6
6	1	III	-	+	-	-	-	+	0.3
7	27	III	-	+	-	-	+	+	0.5
8	1	Ia	+	-	-	+	-	-	1.3
9	5	Ia	+	-	-	-	-	+	0.6

* Equivalent values to 10^6^ CFU for one isolate in each profile; BMEC: bovine mammary epithelial cells

**Table 2:** Comparison of both SCC and biofilm among virulence genes of *S. agalactiae* isolated from cow’s milk with subclinical mastitis.

	SCC * (log10cells/mL)				Biofilm (log10 optical density)		
gene	Positive	Negative	*p*-value		Positive	Negative	*p*-value **
*capIa*	5.98 ± 0.04	5.98 ± 0.05	0.92		−0.39 ± 0.03	−0.38 ± 0.03	0.77
*capIII*	5.98 ± 0.05	5.98 ± 0.04	0.92		−0.38 ± 0.03	−0.39 ± 0.03	0.77
*fbsA*	5.98 ± 0.04	5.98 ± 0.05	0.89		−0.38 ± 0.03	−0.38 ± 0.03	0.99
*fbsB*	5.98 ± 0.05	5.98 ± 0.04	0.89		−0.38 ± 0.03	−0.38 ± 0.03	0.99
*pI2a*	5.97 ± 0.07	5.98 ± 0.03	0.91		−0.39 ± 0.05	−0.38 ± 0.03	0.84
*pI2b*	5.98 ± 0.05	5.98 ± 0.04	0.89		−0.38 ± 0.02	−0.51 ± 0.10	0.19
*hlyB*	5.97 ± 0.03	6.30 ± 0.18	0.10		−0.39 ± 0.02	−0.32 ± 0.13	0.63

* Somatic cell count. ** *p*-value derived from analysis of variance (Tukey test).

## Data Availability

The raw data of this study will be made available by the authors (corresponding author), without reservation, to any qualified researcher.
